# Recent advances in plant and animal genomics are taking agriculture to new heights

**DOI:** 10.1186/s13059-018-1427-z

**Published:** 2018-04-10

**Authors:** Hamid Beiki, Andrea L. Eveland, Christopher K. Tuggle

**Affiliations:** 1 Iowa State University, Ames, IA 50011 USA; 2 Donald Danforth Plant Science Center, St Louis, MO 63132 USA

## Abstract

A report on the International Plant and Animal Genomes (PAG) conference held in San Diego, USA, 13–17 January 2018.

The Plant and Animal Genomes (PAG) meeting, now in its 26th year, is the largest ag-genomics meeting in the world. With 167 different scientific workshops over 6 days covering all major agriculturally important species (as well as endangered species such as the rhinoceros and tiger), any meeting report must be selective. We focus here on research covering comparative analyses, functional genomics, and advances in bioinformatics; however, all poster abstracts and information on both scientific and industry workshops and talks are available from the meeting’s website (http://intlpag.org). From the record-breaking attendance at PAG XXVI of more than 3100 scientists to the breadth of the research highlighted below, we predict that agricultural genomics will continue to accelerate!

## Plenary lectures at PAG XXVI demonstrate the breadth and depth of genomics

Plenary lectures by influential researchers in different areas of plant and animal genetics and genomics provided new perspectives to conference attendees. Gloria Coruzzi (New York University, New York, USA) spoke about temporal transcriptional logic in plant biological responses. She discussed how temporal changes in transcription factor (TF) function can reveal the context for how genomes drive gene regulatory changes. It was interesting to hear how highly transient TF interactions at a target locus (lasting only 10 s) can translate to long-term temporal regulation of the genome. Liang Dong (Iowa State University, Ames, USA) described the application of agricultural sensors and biochips for monitoring cell growth in real time. He explained that recently developed devices can be used to establish a powerful framework for high-throughput precision plant phenotyping. Ed Buckler (United States Department of Agriculture-Agricultural Research Service, Cornell University, Ithaca, USA) presented his vision for “Breeding 4.0” and how to deploy it; for example, how can we distil our knowledge of biological systems to a single number that breeders want? He highlighted various approaches for tapping the genetic potential in crops such as using environmental genome-wide association studies (GWAS) to identify useful alleles from diverse landraces, sequencing many closely related species to predict the most deleterious alleles, and using machine learning to model TF binding preferences and to predict allele-specific expression within and across species. Melissa Wilson Sayres (Arizona State University, Tempe, USA) talked about how aligning all sequences to a single reference genome can produce erroneous results. She demonstrated that her novel software, XYalign, can be used to solve these biases in sex-related experiments by inferring sex chromosome ploidy from next-generation sequencing data. Jay Shendure (University of Washington, Seattle, USA) described three examples of global analyses of development at the whole-organism level through single-cell (sc) RNA-seq, scATAC-seq, and cell-lineage tracing. For the latter, he described molecular tracing of cell lineages in zebrafish. To create such tracers, barcodes containing ten CRISPR–Cas9 cleavage sites were inserted into zebrafish zygotes along with editing reagents. As development proceeded, editing and re-editing of the barcodes created a hierarchy of sites that allowed the derivation of the cell lineages through single-cell sequencing of cell populations later in development. He also described the use of inducible CRISPR–Cas9 constructs to trace late lineages in zebrafish, such as those in the brain. In the final plenary talk, Doreen Ware (United States Department of Agriculture-Agricultural Research Service, Cold Spring Harbor Laboratory, Cold Spring Harbor, USA) described the infrastructure that has been established to analyze plant genomes, especially for maize, and emphasized the need for global cooperation in sharing both data and bioinformatics tools in the era of “big data”. Such cooperation will continue the extraordinary progress made so far in uncovering the links between genotypic variation and phenotypic variation in agricultural plants.

## Animal genomics is set to “go functional”

The Functional Annotation of Animal Genomes (FAANG) Consortium is dedicated to improving genome annotation for domesticated animal species worldwide. The FAANG workshop had 13 speakers who described the current status of FAANG projects in cattle, goat, horse, pig, poultry, sheep, water buffalo, and salmonid species. Several groups presented on early integrative analyses of multi-tissue, multi-datatype data sets to predict chromatin function genome-wide (Jianhua Cao, Huazhong Agricultural University, Wuhan China; Andrea Rau, Institut National de Recherche Agronomique, Jouy en Josas, France; and Huaijun Zhou, University of California Davis, Davis, USA). Amanda J. Chamberlain (Agriculture Victoria, Melbourne, Australia) reported on work in dairy cattle on the genetic control of RNA expression, and she showed that most expression quantitative trait loci (eQTL) are in the same topologically associated domain (TAD) as their target gene and that TADs are more informative than arbitrary distances for linking eQTL with such targets. Other speakers also reported the development of standard laboratory protocols, metadata standards, and data analysis pipelines that are crucial for large-scale integrated analyses across datatypes and species. The critical infrastructure is now in place to fast-track our understanding of domesticated animal genome function.

## Progress on deep genome-wide analysis of vertebrates

A full-day workshop on the Genome 10 K – Vertebrate Genome Project (VGP) was held at PAG 2018. Chaired by Erich Jarvis (Rockefeller University, New York, USA), the morning session featured talks on current best practices and a panoply of new tools for the sequencing and assembly of vertebrate genomes, including a detailed discussion of the value of, and technology needed for, accurate phased genome assemblies. Workshop attendees then discussed the current challenges and approaches for attaining the first phase of the reference VGP, which is sequencing a representative of all orders of vertebrates towards a Phase 3 goal of a genome sequence of all 10,000 vertebrate genera and eventually a Phase 4 goal of all ~ 66,000 species. In the afternoon, talks were presented on current approaches to align and annotate across vertebrates, and the final session covered the nuts and bolts of obtaining permits to share tissue samples across international borders, as well as fundraising and publication plans. It was interesting to hear that the ordinal level Phase 1 of 260 species is being substantially funded through crowd-sourcing among scientists. The outcomes of this inspiring project to understand evolution and, potentially, to provide tools to improve endangered species preservation will be important in both the short term and the long term.

## Accelerating crop productivity through advances in genetics and genomics

This year marks 20 years since the inception of the National Plant Genome Initiative (NPGI), which is dedicated to advancing crop improvement through genome sciences. PAG workshops related to various aspects of plant biology have largely been influenced by this initiative and have highlighted progress towards the enhanced productivity of important food and energy crops through innovations in genomics technologies, high-throughput phenotyping, advanced breeding, and comparative and functional genomics. Examples of how enhanced genetics and genomics resources have been accelerating crop-breeding efforts were highlighted in the Genome-Assisted Breeding workshop. Nils Stein (Leibniz Institut für Pflanzengenetik und Kulturpflanzenforschung, Gatersleben, Germany) reported on genotyping and phenotyping of large, morphologically diverse collections of barley to capture novel genetic variation related to agronomic panicle and seed traits. Yves Vigouroux (Institut de Recherche pour le Développment, Marseilles, France) leveraged resequencing data from 994 diverse pearl millet cultivars and wild accessions to gain insight into the evolutionary history and domestication of this African subsistence and highly drought-tolerant cereal crop. These emerging resources may reveal untapped genetic potential for the improvement of orphan crops.

The workshop on Comparative Genomics highlighted the power of borrowing genomics information across tissues, developmental stages, natural diversity, and closely related species to help define targets for crop improvement. Nathan Springer (University of Minnesota, St Paul, USA) evaluated shared and polymorphic transposable element (TE) insertions between de novo assembled maize genomes and the impact of this variation on nearby genes. John Vogel (Joint Genome Institute, Walnut Creek, USA) suggested that a single plant genome may contain only 50% of the genes in a species when he presented the *Brachypodium* pan-genome project, which included 56 independently annotated genome assemblies. Comparative epigenome analyses that leveraged genome-wide chromatin accessibility maps across tissues and species were presented by Xiaoyu Zhang (University of Georgia, Athens, USA) and Andrea Eveland (Donald Danforth Plant Science Center, St Louis, USA). By annotating conserved and/or divergent functional regions of the genome and integrating informative genomic features (such as TF binding sites, histone modifications, conserved non-coding sequences, and data from GWAS) genetic targets for advanced breeding or precision engineering are prioritized.

The US NPGI workshop was hosted by major funding entities to reflect on the past 20 years of plant genomics research, to consider the effects that funding has had on technological advances for crop improvement, and to discuss focus areas for meeting the grand challenges of the next 20 years. A panel of prominent researchers in diverse areas of plant science presented their visions. Joe Ecker (Salk Institute, La Jolla, USA) emphasized the value of deep, integrative genomics analyses performed at single-cell resolution across model and crop species. Susan McCouch (Cornell University, Ithaca, USA) favored leveraging maximum genetic diversity to accelerate crop improvement and interdisciplinary training. Jan Leach (Colorado State University, Fort Collins, USA) encouraged us to think beyond the plant and to consider the phytobiome at a systems level. Eric Lyons (University of Arizona, Tucson, USA) discussed the need to prepare for unprecedented amounts of data and to train computationally aware scientists. A lively panel discussion that engaged the community on these topics concluded the workshop.

## Emerging bioinformatics tools, techniques, and resources

The goals of animal and plant geneticists are ambitious; for example, one goal is to predict phenotypes by leveraging a deep understanding of genetic and molecular bases of biological processes. This will require improvements to current bioinformatics tools, techniques, and resources. Researchers at PAG XXVI covered a wide range of topics in bioinformatics. Of particular interest, Steven Xijin Ge (South Dakota State University, Brookings, USA) has developed iDEP, a user-friendly web application for exploratory data analysis, differential expression, and pathway analysis of RNA-seq data. The key feature of iDEP is making many powerful R/Bioconductor packages easily accessible by wrapping them under a graphical interface. Anthony Greenberg (Bayesic Research, Ithaca, USA) has developed memory-efficient sampling software, sampleSNPs, to generate representative samples from large DNA sequence variation data sets. This software can be very useful when a quick test is needed on computationally intensive pipelines. Fábio Mendes (Indiana University, Bloomington, USA) reported on a stochastic model, CAFE, to identify gene gain and losses through comparative genomic analyses. The main feature of this model was its robustness in the face of fragmented and incomplete assemblies. Shuhui Song (BGI, Shenzhen, China) has developed the GVM, a public repository of genomic variation and genotype association data for a wide range of plants and animals. This user-friendly data repository can be helpful for gaining a better understanding of population genetic diversity and for deciphering complex mechanisms associated with different phenotypes. Jérémy Berthelier (Institut Français de Recherche pour l’Exploitation de la Mer, Nantes, France) has developed a new bioinformatics pipeline, PiRATE, for the full-length detection and annotation of TE families in non-model organisms. The main advantage of PiRATE is its applicability to poorly studied genomes, as it is not dependent on the databank of known TEs. The National Center for Genome Analysis Support (Indiana, USA) has developed Jetstream, a cloud computing environment. The goal of Jetstream is to assist researchers who have no computational background to run their analyses on high-performance computing systems. This project also assists researchers who are interested in robust transcriptome assembly by creating a workflow template using four software packages (Trinity, SOAP-denovo, TransABySS, and VelvetOases). Elizabeth Tseng (Pacific Biosciences, Menlo Park, USA) described novel software, IsoPhase, that can be used to retrieve allele-specific isoform information from PacBio long-read transcript data. Validation of IsoPhase-called haplotypes by phased assembly from an F1 of Angus X Brahman cattle showed the potential of this software for revealing allelic imbalance in isoform expression. Haixiao Dong (Washington State University, Pullman, USA) proposed a Bayesian method for the prediction of hybrid performance based on principal component analysis (PCA). The main feature of this method is its computational speed (by reducing the number of SNPs using PCA), which is a limiting factor in many laboratories. Jens Keilwagen (Julius Kühn-Institut, Quedlinburg, Germany) presented an extension of the gene prediction tool GEMOMA that utilizes amino acid sequence conservation, intron position conservation, and RNA-seq data for homology-based gene prediction. This approach can be useful in genome annotation pipelines.

## Conclusion

The 2018 PAG XXVI conference provided an excellent opportunity for open discussions on how to tackle the current challenges in plant and animal genomics. The presentations described above provided a snapshot of the tremendous advancements in many areas of both basic and applied genomics, as well as in the continued development of useful bioinformatics tools to best explore these ever-expanding data sets. In 2019, PAG XXVII is expected to continue this trend, and this trajectory bodes well for increased understanding and utilization of nature’s bounty.

## Abbreviations

FAANG: Functional Annotation of Animal Genomes
PAG: Plant and animal genome
TE: Transposable element
TF: Transcription factor

